# The Effect of Self-Help Mindfulness-Based Stress Reduction Exercise Therapeutics on the Psychological Status and Sleep Quality of Hubei Medical Staff

**DOI:** 10.31083/AP44140

**Published:** 2025-06-23

**Authors:** Qian Yin, Chenxi Shi, Shuqin Wang

**Affiliations:** ^1^Department of Neurology, Beijing Chao-Yang Hospital, Capital Medical University, 100020 Beijing, China; ^2^Department of Respiratory and Critical Care Medicine, Beijing Chao-Yang Hospital, Capital Medical University, 100020 Beijing, China

**Keywords:** coronavirus disease 2019, medical staff, mindfulness-based stress reduction, psychological status, sleep quality

## Abstract

**Objective:**

To explore the effect of self-help mindfulness-based stress reduction exercise therapeutics (MBSRET) on the psychological status and sleep quality of medical staff.

**Method:**

A total of 85 Hubei medical staff were randomly divided into an intervention group (n = 43) and a control group (n = 42). The intervention group received MBSRET for 8 weeks and the control group received routine care. Psychological status and sleep were assessed using the Symptom Checklist-90 (SCL-90), the Perceived Stress Scale (PSS), and the Pittsburgh Sleep Quality Index (PSQI) before and after the intervention.

**Result:**

After the intervention, SCL-90, PSS, and PSQI scores were significantly lower in the control group (*p* < 0.001 for all), indicating that MBSRET could effectively improve the psychological status and sleep quality of Hubei medical staff.

**Conclusions:**

Mindfulness-based stress reduction exercise therapy is a simple, convenient, and low-cost intervention method that can be widely applied to improve the mental health care of medical staff during public health emergencies.

**Clinical Trial Registration:**

The study was registered at https://www.isrctn.com/ISRCTN84911422, registration number: ISRCTN84911422.

## Main Points

1. Medical staff are highly receptive to mindfulness-based stress reduction exercise therapy (MBSRET).

2. Mindfulness-based stress reduction exercise therapy can effectively improve the psychological status and sleep quality of the medical staff who supported the coronavirus disease 2019 pandemic prevention and control in Hubei Province.

3. This study provides novel and valuable evidence for applying MBSRET in mental health care for medical staff during public health emergencies.

## 1. Introduction

The COVID-19 pandemic has become a global public health emergency, affecting more than 200 countries and regions, and causing more than 4 million deaths as of December, 2023 [1]. It was declared a pandemic on March 11, 2020 [2].

To curb the spread of COVID-19, China launched a nationwide mobilization of medical resources and personnel to support prevention and control efforts in Hubei Province [3]. The medical staff (doctors and nurses) faced enormous challenges and pressures: the high risk of infection, heavy workload, long working hours, lack of protective equipment, isolation from family and friends, exposure to death and suffering, stigma, and discrimination [4]. These factors can lead to anxiety, depression, and insomnia [5]. The rates of psychological distress (34% to 76%) and insomnia (34% to 61%) were both high among medical staff during the COVID-19 pandemic [6, 7]. These psychological problems can impair physical and mental health, work performance, and quality of life and they can increase the risk of medical errors and adverse events [8]. Therefore, it is essential to provide effective psychological interventions for medical staff who supported the pandemic prevention and control in Hubei Province to help them cope with stress and improve their mental health and well-being [9].

Considering the limited time and resources, it is difficult to implement conventional psychological interventions (e.g., in-person counselling or pharmacotherapy) for medical staff [10]. Moreover, some medical staff may be reluctant to seek professional help due to stigma, privacy concerns, or accessibility issues [11]. Therefore, there is a need for alternative, low-cost psychological interventions that are simple, convenient, self-help-oriented, and easily integrated into the daily routines of medical staff.

A potential psychological intervention that meets the criteria is mindfulness-based stress reduction exercise therapy (MBSRET), a self-help intervention that combines mindfulness and exercise to enhance physical and mental health [12]. Mindfulness is a state of awareness and attention to the present moment through an open, curious, and non-judgmental attitude [13]. Physical exercise can enhance or maintains physical fitness, whereas mindfulness helps individuals regulate their emotions by reducing negative thoughts and increasing positive mood [14]. Physical exercise can help individuals improve their cardiovascular, respiratory, musculoskeletal, and immune systems and releases endorphins, which are natural painkillers and mood enhancers [15]. Mindfulness-based stress reduction exercise therapy can be performed by individuals according to their preferences (such as body scan, yoga, sitting meditation) [16]. As is well known, meditation helps to soothe emotions. Meditation is also helpful for mental health among medical staff. Research has indicated that mindfulness meditation interventions are advantageous in supporting the mental health of medical staff [17].

Several studies have demonstrated the effectiveness of MBSRET in improving the psychological status and sleep quality across various age groups and disease backgrounds [18, 19]. Research has indicated that mindfulness-based training can improve the psychological health of nurses, but there is a lack of conclusive evidence [20]. Therefore, this study aims to explore the effect of MBSRET on the psychological status and sleep quality of medical staff and to provide evidence for its application in mental health care for medical staff during public health emergencies.

## 2. Methods

### 2.1 Participants

The participants of this study were medical staff who were sent to support pandemic prevention and control in Hubei Province between January and April, 2020. Inclusion criteria: (1) over 18 years old and (2) able to access the internet and use the MBSRET mobile application. Exclusion criteria: (1) having a history of mental illness or receiving psychological treatment, (2) having a physical condition that prevents performing physical exercise, and (3) being pregnant or lactating. Ninety-seven medical staff were dispatched to Hubei, and Beijing Chaoyang Hospital of Capital Medical University conducted centralized training before their departure. We recruited them at this time. We provided a detailed introduction to this study and invited them to participate. Nine people refused to participate and 87 people agreed to participate. After screening, 85 people met the requirements of this study and were included. They were randomly assigned to either the intervention group (n = 43) or the control group (n = 42) using a computer-generated random number table. Members of the research team (researchers) were responsible for recruitment, randomization, intervention, and evaluation. In order to avoid the influence of the subjective will of the researchers, those responsible for the evaluation did not participate in recruitment, randomization, intervention or other work, which were completed by other researchers. Therefore, this study was a single-blind study. The study was approved by the Ethics Committee of Beijing Chao-Yang Hospital, Capital Medical University (Approval No: 2024-K-869) and adhered to CONSORT guidelines. The study was conducted in accordance with the Declaration of Helsinki. All participants provided signed informed consent.

### 2.2 Intervention

The intervention group received MBSRET for 8 weeks and the control group received routine care. The MBSRET intervention consisted of two components: mindfulness and physical exercise. The mindfulness component was based on the MBSR program, a well-established and evidence-based intervention for various psychological and physical problems. It includes 8 weekly meetings, each lasting for 2.5 hours, and a single-day retreat. The program taught various mindfulness practices: body scanning, mindful breathing and movement, sitting meditation, and loving-kindness meditation. The participants were instructed to practice these exercises at home or work for at least 30 minutes every day, 6 days per week, with the guidance of the MBSRET mobile application. The mobile application was developed by our research team based on the MBSR curriculum and existing mindfulness applications. It provided audio recordings, videos, text, and images to guide the participants through the exercises. The application also recorded the participants’ compliance, duration of exercise, and feedback.

The physical exercise component was based on the American College of Sports Medicine (ACSM) guidelines for physical activity and health [21]. The ACSM guidelines suggest that adults should perform at least 150 minutes of moderate-intensity aerobic exercise, 75 minutes of vigorous-intensity aerobic exercise, or a combination of both each week. Further recommendations included performing muscle-strengthening activities involving all the major muscle groups at least 2 days per week. The participants were instructed to choose their types and modes of physical exercise according to their preferences, abilities, and schedules. They were advised to perform physical exercise for at least 30 minutes each day, 5 days per week, at a moderate-to-vigorous intensity level with the guidance of the MBSRET mobile application. The mobile application provided various options, suggestions, and tips for physical exercise; it also recorded the participants’ compliance, duration and intensity of exercise, and feedback.

The control group received routine care, it included the standard medical care and psychological support provided by the local health authorities and hospitals. They did not receive any specific interventions or instructions regarding mindfulness or physical exercise. They were also asked to use the MBSRET mobile application, but only to complete the outcome measures and provide basic demographic and clinical information.

Participants use diary cards to record the intervention measures they received, and we promptly reminded them to accept the intervention and evaluate their treatment fidelity through the diary cards.

### 2.3 Measures

The outcome measures of this study were the psychological status and sleep quality of the participants. Psychological status was assessed using the Symptom Checklist-90 (SCL-90), the Perceived Stress Scale (PSS), and the Pittsburgh Sleep Quality Index (PSQI).

#### 2.3.1 Symptom Checklist-90 

The SCL-90 is a questionnaire that measures nine dimensions of psychological symptoms [22]. This study used the Chinese version [23]. The participants were asked to rate the extent to which they experienced each symptom in the past week on a 5-point Likert scale, ranging from 0 (not at all) to 4 (extremely). The score is directly proportional to the severity of the symptoms. The SCL-90 demonstrated high reliability and validity, as evidenced by Cronbach’s alpha coefficient ranging from 0.77 to 0.90 for the nine dimensions and 0.97 for the Global Severity Index (GSI).

#### 2.3.2 Perceived Stress Scale 

The PSS is a 10-item self-reporting questionnaire designed to gauge individuals’ perception of stress levels in their life situations over the past month [24]. This study used the Chinese version [25]. The participants were asked to rate how often they felt or thought a certain way on the 5-point Likert scale, ranging from 0 (never) to 4 (very often). The score is directly proportional to the severity of psychological symptoms. The PSS showed a Cronbach’s alpha coefficient of 0.86.

#### 2.3.3 Pittsburgh Sleep Quality Index 

The PSQI is a 19-item self-reporting questionnaire that measures the quality and patterns of sleep in the past month [26]. This study used the Chinese version [27], which consists of 17 parts, including sleep quality and sleep duration, etc. Score is inversely proportional to sleep quality. The PSQI showed a Cronbach’s alpha coefficient of 0.83. The outcome measures were administered to participants before and after the intervention using the MBSRET mobile application. Participants were directed to complete the measures at their convenience, ensuring honesty and accuracy in their responses. Reminders to complete the assessments were provided through the MBSRET app and by research assistants via phone calls or text messages.

### 2.4 Data Analysis

Data analysis was performed using SPSS 26.0 software (IBM, Armonk, NY, USA). Mean, standard deviation, frequency, and percentage were used to describe the demographic and clinical characteristics of the participants. The student’s *t*-test was used for data that conformed to normal distribution. Categorical data were assessed using Pearson’s chi-squared test, Fisher’s exact test, and the Fisher–Freeman–Halton test. The repeated measures analysis of variance (ANOVA) method was used to compare changes in the outcome measures between the two groups over time. The effect size was calculated using partial eta squared (ηp^2^), representing the proportion of variance in the outcome measures explained by the intervention. The level of statistical significance was set at *p* < 0.05.

## 3. Results

### 3.1 Demographic Characteristics

Table 1 shows the demographic characteristics of the participants. There were no significant differences between the two groups regarding age, sex, marital status, educational level, professional position, and working years (*p* > 0.05).

**Table 1.** **Demographic and clinical characteristics of the participants**.

Variable		Intervention Group (n = 43)	Control Group (n = 42)	*p*-value
Age (years)		33.21 ± 7.46	33.29 ± 7.59	0.963
Sex				0.955
	- Female	34 (79.1%)	33 (78.6%)	
	- Male	9 (20.9%)	9 (21.4%)	
Marital status				1.000
	- Married	39 (90.7%)	38 (90.5%)	
	- Unmarried	4 (9.3%)	4 (9.5%)	
Education level				0.986
	- Secondary vocational	8 (18.6%)	8 (19.0%)	
	- Junior college	22 (51.2%)	22 (52.4%)	
	- Bachelor’s degree and above	13 (30.2%)	12 (28.6%)	
Position				0.799
	- Doctor	7 (16.3%)	6 (14.3%)	
	- Nurse	36 (83.7%)	36 (85.7%)	
Professional title				1.000
	- Primary	19 (44.2%)	18 (42.9%)	
	- Intermediate	21 (48.8%)	22 (52.4%)	
	- Associate senior and above	3 (7.0%)	2 (4.7%)	
Working years		7.14 ± 2.31	7.10 ± 2.24	0.931

### 3.2 Compliance

Adherence to the intervention was assessed by recording the number and duration of mindfulness and physical exercise sessions completed by participants in the intervention group, as documented by the MBSRET mobile application. The results revealed that during the 8-week intervention period, the intervention group completed an average of 39.12 ± 7.83 mindfulness sessions and 34.67 ± 6.54 physical exercise sessions, each lasting for an average duration of 32.45 ± 5.12 minutes and 31.78 ± 4.87 minutes, respectively. The adherence rate was computed by dividing the number of completed sessions by the number of prescribed sessions and then multiplying by 100%. For the intervention group, the adherence rate was 81.50% ± 16.31% for the mindfulness component and 72.23% ± 13.65% for the physical exercise component.

### 3.3 Changes in Psychological Status

Table 2 shows changes in psychological status, as measured by the SCL-90 and the PSS. A significant interaction effect between group and time for the SCL-90 and PSS scores was revealed by repeated measures ANOVA (*p* < 0.05), suggesting distinct changes in psychological status over time between the intervention and control groups. The intervention group had significantly lower scores in the SCL-90 and the PSS after the intervention compared with before the intervention (*p* < 0.05), and the control group had no significant changes in the SCL-90 and the PSS scores over time (*p* > 0.05). Additionally, after the intervention, the intervention group had significantly lower scores in the SCL-90 and the PSS (*p* < 0.05). There was no significant difference between the two groups before the intervention (*p* > 0.05). ηp^2^ showed that the effect sizes of the intervention were large for the SCL-90 and the PSS scores (see Table 2).

**Table 2.** **Changes in psychological status as measured by the SCL-90 and PSS**.

Measure	Group	Time	Mean ± SD	*p*	ηp^2^
SCL-90	Intervention	Pre	1.62 ± 0.35	<0.001	0.38
		Post	0.98 ± 0.24		
	Control	Pre	1.59 ± 0.33	0.928	0
		Post	1.58 ± 0.32		
	Group × Time			<0.001	0.37
PSS	Intervention	Pre	23.67 ± 4.56	<0.001	0.34
		Post	14.21 ± 3.45		
	Control	Pre	23.12 ± 4.32	0.632	0
		Post	22.86 ± 4.28		
	Group × Time			<0.001	0.33

Note: SCL-90, Symptom Checklist-90; PSS, Perceived Stress Scale; SD, Standard Deviation; ηp^2^, Partial Eta Squared.

Changes in sleep quality, as measured by the PSQI, are shown in Table 3. There was a significant interaction effect between group and time for the PSQI score by repeated measures ANOVA (*p* < 0.05), indicating that the intervention and the control groups had different changes in sleep quality over time. The intervention group had a significantly lower score on the PSQI after the intervention (*p* < 0.05). The control group had no significant change in the PSQI score over time (*p* > 0.05). Additionally, after the intervention, the intervention group also had a significantly lower score on the PSQI (*p* < 0.05). There was no significant difference between the two groups before the intervention (*p* > 0.05). ηp^2^ showed that the effect size of the intervention was large for the PSQI score (see Table 3).

**Table 3.** **Changes of sleep quality as measured by the PSQI**.

Measure	Group	Time	Mean ± SD	*p*	ηp^2^
PSQI	Intervention	Pre	9.67 ± 2.45	<0.001	0.31
		Post	5.12 ± 1.67		
	Control	Pre	9.43 ± 2.32	0.741	0
		Post	9.38 ± 2.28		
	Group × Time			<0.001	0.3

Note: PSQI, Pittsburgh Sleep Quality Index.

## 4. Discussion

This study aimed to explore the effect of MBSRET on the psychological status and sleep quality of the medical staff who were sent to support the COVID-19 pandemic prevention and control in Hubei Province. The results showed that MBSRET could effectively improve the psychological status and sleep quality of the medical staff.

This study demonstrates the effectiveness of mindfulness and exercise, which is consistent with previous studies [21]. However, this is the first study to investigate the effect of MBSRET on the medical staff who supported the COVID-19 pandemic prevention and control in Hubei Province. It provides novel and valuable evidence for the application of MBSRET in the field of mental health care for medical staff during public health emergencies.

Constructive response measures are beneficial [28]. The mechanisms of MBSRET that improved the psychological status and sleep quality of the medical staff were multifaceted and interrelated, involving emotional, cognitive, physiological, neurobiological, behavioral, and lifestyle aspects [29]. This intervention method could enhance the regulation of emotions and cognition, which are essential skills for coping with stress and maintaining mental health [30]. The intervention can also help break the cycle of rumination, worry, and catastrophizing, allowing the adoption of a more adaptive and flexible perspective on challenges and difficulties [30]. By modulating the physiological and neurobiological responses to stress, MBSRET could improve the cardiovascular, respiratory, musculoskeletal, and immune systems, as well as sleep architecture and quality [31]. It could also influence the brain structures and functions that are related to stress, emotions, and cognition, as well as enhance neural connectivity and plasticity [32].

The highlights of this study were its randomized controlled design, its large sample size, the use of valid and reliable outcome measures, the use of the MBSRET mobile application, and the high adherence rate of the intervention group. However, some limitations should be acknowledged and addressed in future studies. First, this study did not have a long-term follow-up; therefore, the durability and maintenance of the intervention effects are unknown. This study lacked monitoring of participant compliance, as well as compliance and consistency in the implementation of the protocol and intervention measures, which may have led to bias in the results. Second, this study did not include any objective measures of the psychological and physiological outcomes, which can lead to bias due to the use of self-reporting questionnaires. Third, this study did not control for potential confounding factors: baseline differences, co-interventions, social support, and expectation effects. Fourth, as most nurses were women, the participants included in this study were mostly women, which may limit the representativeness of the results.

Therefore, future studies should conduct a longer-term follow-up and use more comprehensive measures to assess the effects and mechanisms of MBSRET. In addition, more rigorous methods to control for biases and to ensure the internal validity of the study should be considered: the use of stratified randomization, placebo or active control group, the blinding or masking technique, and covariate analysis.

## 5. Conclusions

In conclusion, this study showed that MBSRET could effectively improve the psychological status and sleep quality of the medical staff who supported the COVID-19 pandemic prevention and control in Hubei Province. This low-cost intervention method is simple and convenient and can be widely applied to improve the mental health care of medical staff during public health emergencies. Future studies should further explore the mechanisms, outcomes, and applications of MBSRET and provide more evidence and guidance for the mental health care of medical staff during public health emergencies.

## Data Availability

The datasets used and/or analyzed during the current study are available from the corresponding author on reasonable request.
